# Peripheral patellar denervation has a better effect in reducing postoperative anterior knee pain than patellar resurfacing in TKA

**DOI:** 10.1097/MD.0000000000031584

**Published:** 2022-11-11

**Authors:** Xiaohui Ji, Xiaodan Huang, Yingying Zhang, Ming Zhao, Yaming Liu, Yanxin Cheng

**Affiliations:** a Department of Orthopedics, Cangzhou Hospital of Integrated TCM-WM, Cangzhou, Hebei, P.R. China; b Department of Orthopedics, the Third Hospital of Hebei Medical University, Hebei, P.R. China; c Department of pain, the Third Hospital of Hebei Medical University, Hebei, P.R. China.

**Keywords:** anterior knee pain, patellar denervation, patellar resurfacing, total knee arthroplasty

## Abstract

Patellar resurfacing (PR) and peripheral patellar denervation (PD) are common surgical treatments for knee osteoarthritis (KOA) in total knee arthroplasty (TKA). The aim of study was to compare preventive effect on postoperative anterior knee pain (AKP) between PR and peripheral PD in TKA. A total of 202 patients who underwent unilateral TKA were randomized into 3 groups: T, TPD, and TPR. Patients in T group received simple TKA, patients in TPD group received TKA combined PD while patients in TPR group received TKA combined PR. Incidence, intensity, and presentation time of AKP and clinical outcomes were evaluated at 3, 6, 9, 12, 18, and 24 months postoperatively. The incidence of AKP was significantly lower and the intensity of AKP and patients’ satisfaction score were significantly better at 3 months after surgery in group TPD and TPR compared with group T. Compared with group TPR, the intensity of AKP was significantly better at 3 months after surgery in group TPD. There were no significant difference in Oxford knee score, range of motion (ROM), patellar score, knee society score (KSS) and activities of daily living (ADL) score among 3 groups in the follow-up period. Both PD and PR can effectively reduce the intensity and incidence of AKP after TKA and improve patients’ satisfaction at 3 months after TKA. Additionally, PD is more effective on alleviating AKP than PR.

## 1. Introduction

With the increasing trend of population aging, more and more elderly people are troubled by knee osteoarthritis (KOA).^[1]^ KOA is a degenerative disease in the middle-aged and elderly, which is caused by cartilage degeneration, fibrosis, wear and tear, subchondral bone sclerosis, cystic change, osteophyte formation at the edge of the joint, synovitis hyperplasia, and then leads to joint capsule and ligament contracture.^[2]^ As KOA developed, the pain of knee joint is obvious and the activity is seriously limited. X-ray shows multiple osteophyte hyperplasia in the joint, and the gap is narrow or even disappears. Severe cases may be accompanied by varus or valgus deformity. End-stage KOA seriously affects the health and quality of life of middle-aged and elderly people, causing a huge economic burden to patients and society.^[3,4]^ Compared with traditional conservative treatment, knee arthroplasty has become one of the important means to solve end-stage KOA, especially total knee arthroplasty (TKA). TKA can relieve patients’ joint pain and dysfunction to the greatest extent, and restore joint function.^[5,6]^

In light of the maturation of TKA and prosthetic design technologies, more and more people choose TKA to treat end-stage KOA. Zhou et al have confirmed that the annual average growth rate of TKA surgery volume in China from 2009 to 2015 was more than 20%.^[7]^ However, a retrospective study of 15 years after TKA showed that 20% of patients were dissatisfied with TKA, and one of the main reasons was the occurrence of anterior knee pain (AKP).^[8]^ In TKA operation, the injury of soft tissue around patella, such as ligament, synovium and infrapatellar fat pad, is the cause of AKP. Intraoperative nerve compression and traction are also related to the occurrence of AKP. AKP after TKA will seriously affect patients’ daily activities (such as walking, climbing stairs, cycling, standing up, etc), especially for Asians who are used to sitting cross legged.^[9,10]^ Therefore, it is urgent to explore effective solutions to AKP after TKA.

For APK after TKA, many clinicians and medical scientists have made many preventive explorations in animal models and clinical practice, including patellar resurfacing (PR)^[11]^ and peripheral patellar denervation (PD).^[12]^ Thiengwittayaporn S et al have confirmed that peripheral PD (electric cautery to remove the peripheral patellar nerve, the depth of electric cautery is 2~3 mm, and the range of denervation is 4~8 cm) can effectively reduce the incidence of AKP after TKA and improve the activity function of the knee joint.^[12]^ Chen K et al conducted a meta-analysis including Thirty-two trials and 6887 knees to compare the outcomes of PR and nonresurfacing in TKA. The results showed that PR could reduce the occurrence of reoperation and noise after surgery, as well as increase the knee society score (KSS) and function score, while it might not influence the outcomes such as AKP, ROM, Oxford score, KOOS, visual analogous scale (VAS), Feller score, patellar tilt and the patients’ satisfaction. Therefore, TKA combined with PR and peripheral PD are effective interventions to reduce AKP after TKA. However, there is still no research to compare the above 2 preventive measures of AKP.

Given that, the aim of this study was to compare preventive effect on postoperative AKP between PR and simple peripheral PD in TKA, so as to provide a theoretical basis for making a more comprehensive treatment strategy for patients with KOA.

## 2. Materials and Methods

This prospective, randomized, controlled study was designed in accordance with the principles outlined in the Declaration of Helsinki. This study has been approved by the institutional review board of the Third Hospital of Hebei Medical University and all participants have signed informed consent forms.

### 2.1. Inclusion and exclusion criteria

From July 2017 and January 2020, patients underwent unilateral TKA in the Third Hospital of Hebei Medical University were enrolled. The inclusion criteria were as follows: Osteoarthritis, the diagnosis meets the diagnostic criteria of osteoarthritis determined by the American rheumatology society, and it is ineffective after conservative treatment; The pathological degree of the knee joint on the operative side was basically the same, and both reached a severe degree; The clinical and imaging data of participants were complete; Preoperative American Society of Anesthesiologists physical classification: Ⅰ to Ⅲ. The exclusion criteria were as follows: History of infectious arthritis and osteomyelitis; History of patellar fracture or patellofemoral joint surgery; Have a history of tibial osteotomy, artificial hip replacement or patella fracture; Lower limb deformity and restricted movement caused by other reasons; Patients with cerebral infarction, Parkinson’s disease, Alzheimer’s disease, psychosis, etc, which affect postoperative rehabilitation exercise and follow-up compliance; Range of motion (ROM) are less than 90° or flexion contracture are more than 30°. Then patients were randomly assigned into 3 groups with random number table: TKA group (group T), TKA combined with PR (group TPR) and TKA combined with PD (group TPD).

### 2.2. Operating and anesthesia method

With the PS total knee system (Legion PS Total Knee System, Smith & Nephew, Memphis, TN), all procedures were carried out utilizing a minimally invasive method. Every patient underwent standard intraoperative monitoring, such as oxygen saturation, electrocardiogram, and noninvasive blood pressure measurements and was given general anesthesia during the procedure. Patients were given propofol, midazolam, and fentanyl/sufentanil to complete anesthesia induction and received laryngeal mask placement and mechanical ventilation. Controlling BIS 40 to 60 allowed for the preservation of anesthetic depth. Palonosetron (0.25 mg) were administered to combat postoperative nausea and vomiting.

**Figure 1.**
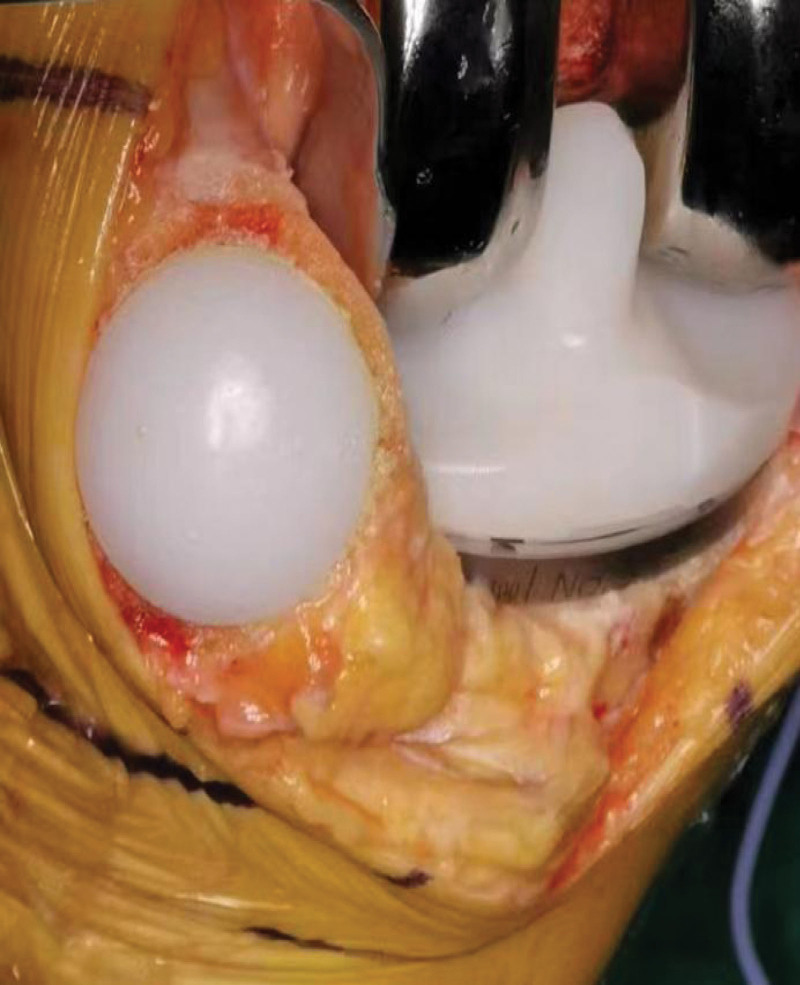
Surgical procedure of patellar resurfacing.

**Figure 2.**
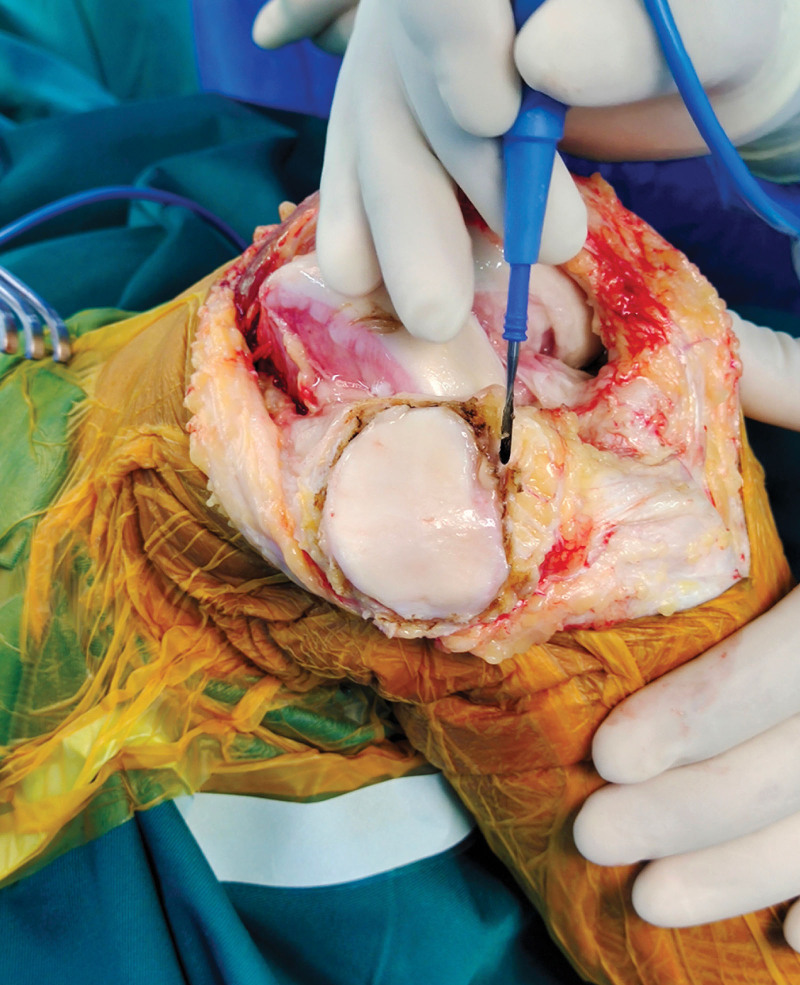
Surgical procedure of patellar denervation.

All TKAs were done using an anterior midline longitudinal skin incision and a medial parapatellar arthrotomy. The front of the patella is cut through after the incision is made on the proximal side of the kneecap. As for the medial edge of the tibial nodule, the skin, subcutaneous and fascia are cut in turn, along the midline of the quadriceps tendon to the superior, and then along the medial side of the tibial nodule along the medial edge of the patellar ligament. The knee cavity can be seen by opening the patella. The knee was bent at 90 degrees and the joint capsule was stripped, thereby extensively exposing the interior of the knee. The joint cavity was cleaned, the medial-lateral meniscus, proliferating osteophytes, anterior cruciate ligament, and partial subpatellar fat pads were removed, and the soft tissue around the knee. All prostheses were used with no retained posterior ACL type. For TKA, the technique utilizing intramedullary femoral and extramedullary tibial alignment guides was followed. Choose the proper prosthesis when the osteotomy is finished. The knee was bent to 90 degrees without dislocating after all knee prosthetics had been fixed in place. the cement was fixed, the knee was bent and extended again, and the knee alignment, lateral collateral ligament tension and the patella movement trajectory were checked. Patients in group TPR received additional PR: Following the measurement and recording of the patellar thickness, the osteotomy thickness was calculated. Symmetrical osteotomy was performed with a patellar guide, requiring the reserved patellar thickness of at least 12 mm after the osteotomy. The patella prosthesis was installed and the patella thickness was measured again, requiring the displaced patella to return to its original thickness (Fig. 1). Additional PD was administered to patients in group TPR: resurface the patella’s articular surface, remove extra soft tissue and proliferative osteophytes, and conduct clock type electric cautery around the patella to denervate with high-frequency electric knife, electric cautery is controlled at a depth of around 3mm (Fig. 2). After all surgical operations, tourniquet was loosen and thumb test was performed to determine whether the patella movement track is good after washing with a large amount of normal saline. Stop bleeding tightly, count the instruments and gauze without error, routinely place the drainage tube, close the incision layer by layer, and press and bind with sterile dressing. Following surgery, patient-controlled intravenous analgesia was frequently used.

Patients were instructed to undertake isometric quadriceps workouts and straight-leg lifts immediately after surgery. After surgery, patients are encouraged to walk with a walker and do active and passive ROM exercises twice a day for the first 6 weeks. Patients were encouraged to walk unassisted after 2 to 3 weeks after surgery.

### 2.3. Data collection

After surgery, patients were evaluated at 3, 6, 9, 12, 18, and 24 months. An evaluation of the incidence, intensity, and presentation time of AKP, ROM, KSS, Oxford knee score, patellar score, activities of daily living (ADL) score, patient satisfaction score, and VAS of overall knee pain was performed by another clinician blinded to the trial intervention. A goniometer was used to measure the active knee flexion and extension angles of the patient while supine. Three months postoperative radiographic parameters were evaluated by 2 independent investigators who were blinded to trial interventions using a picture archive and communication platform. Radiographs taken anteroposteriorly were used to measure the tibiofemoral angle. Congruency of the patella was assessed using Merchant view and measured by lateral patellar tilt, displacement of the patella from the midline of the trochlea patellar displacement and patellar shift index.

### 2.4. Statistical analysis

#### 2.4.1. Sample size calculation.

After simple TKA, approximately 20% of patients developed AKP.^[8]^ We hypothesized that the incidence of AKP would decrease from 20% to 10% after TKA combined with PR or PD. A minimum sample size of 60 subjects per group or 180 subjects overall was required with a significance of 0.05 and a strength of 0.9 (PASS 15.1). Based on a 10% abscission rate, the sample size was 66.

#### 2.4.2. Outcome analysis.

All statistical analysis was performed with the Statistical Package for Social Sciences software (version 23.0; SPSS Inc., Chicago, IL). The continuous data were expressed as mean ± standard deviation (SD) or median (interquartile range, IQR). The demographics and preoperative data of 3 groups were compared using the independent *t* test (Student *t* test) and chi-squared test. An independent *t* test and the chi-squared test were used to compare clinical and radiographic outcomes between 3 groups. A *P* value of .05 indicates a statistical difference.

## 3. Results

### 3.1. Subject characteristics

**Figure 3.**
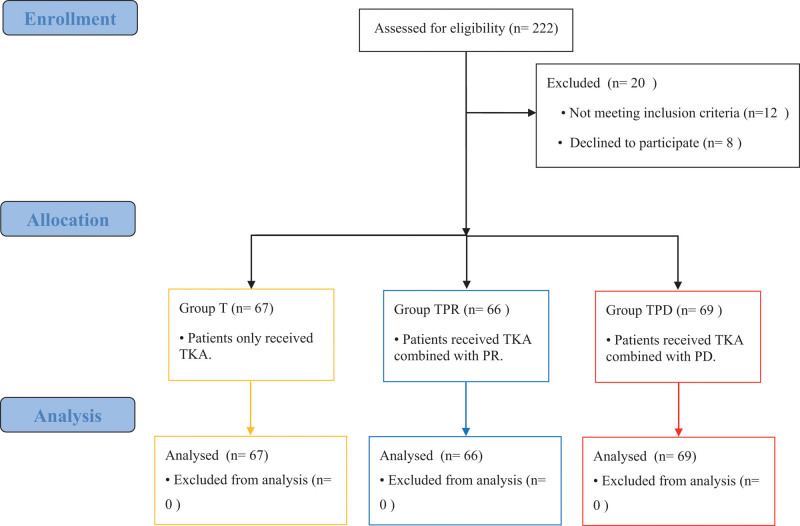
Flow chart of the study. Group T: yellow; Group TPR: blue; Group TPD: red. PD = patellar denervation, R = Patellar resurfacing.

In total, 222 eligible patients were enrolled from July 1, 2018 to December 31, 2021. Among them, 12 patients did not meet the inclusion criteria, 8 patients refused to participate this study. Finally, 202 patients were randomly assigned into 3 groups: TKA group (group T, n = 67), TKA combined with PR group (group TPR, n = 66) and TKA combined with peripheral PD group (group TPD, n = 69) (Fig. 3). As shown in Table 1, there were no statistically significant differences among the 3 groups with respect to age, body mass index, sex, ASA physical status, surgical site, surgical time and follow-up time.

**Table 1:** Baseline characteristics among 3 groups.

Variables	Group T (n = 67)	Group TPR (n = 66)	Group TPD (n = 69)	*χ*^2^ value	*P* value
**Age (yrs**)	67.2 ± 6.4	65.1 ± 6.0	66.3 ± 6.2	0.234	.534
**Gender**				0.297	.465
Male	32	35	33		
Female	35	31	36		
**BMI (kg/m^2^**)	27.6 ± 2.8	28.3 ± 3.0	28.1 ± 2.9	0.202	.664
**ASA**				0.221	.573
Ⅰ	10	8	8		
Ⅱ	56	58	61		
Ⅲ	1	0	0		
**Surgical side**				0.207	.657
Right	38	35	33		
Left	29	31	36		
**Surgical time** **(mins**)	43.6 ± 8.8	48.9 ± 10.2	45.3 ± 9.6	0.459	.193

ASA = American society of anesthesiologists, BMI = body mass index.

### 3.2. Comparison of radiographic evaluation results of knee joint

After 3 months of follow-up, radiographic evaluation showed there were no significant difference among 3 groups regarding tibiofemoral angle, lateral patellar tilt, patellar displacement and patellar shift index (Table 2).

**Table 2:** Comparison of radiographic evaluation results among 3 groups.

Variables	Group T (n = 67)	Group TPR (n = 66)	Group TPD (n = 69)	F value	*P* value
Tibiofemoral angle (°)	6.3 ± 3.7	6.7 ± 4.0	6.2 ± 3.5	0.428	.481
Lateral patellar tilt (°)	7.5 ± 2.3	7.7 ± 2.4	7.5 ± 2.2	0.362	.687
Patellar shift index	2.0 ± 0.1	1.9 ± 0.2	1.8 ± 0.1	0.355	.696
Patellar displacement (mm)	4.5 ± 0.3	4.1 ± 0.2	4.3 ± 0.3	0.413	.473

### 3.3. Comparison of postoperative AKP related index at different follow-up time

In all 3 groups, AKP presented at 3 months postoperatively. Compare with group T, the incidence of AKP was significantly lower (TPR: t = 1.942, *P* = .031; TPD: t = 3.037, *P* = .001) and the intensity of AKP was significantly better at 3 months after surgery (TPR: t = 2.969, *P* = .002; TPD: t = 2.354, *P* = .011) but not at the later follow-up time points in group TPR and TPD. Compared with group TPR, the intensity of AKP was significantly better at 3 months after surgery in group TPD (t = 2.392, *P* = .009) and there was no difference in the incidence of AKP between 2 groups (Table 3).

**Table 3:** Comparison of postoperative AKP related index at different follow-up time among 3 groups.

Variables	Group T (n = 67)	Group TPR (n = 66)	Group TPD (n = 69)	F value	*P* value
**Incidence of AKP (%**)	11 (16.42%)	4 (6.06%)	5 (7.24%)	113.6	.021
**Intensity of AKP**					
3 mo	54.23 ± 12.3	19.2 ± 8.7	11.2 ± 7.6	168.7	.013
6 mo	18.7 ± 7.9	15.4 ± 6.83	10.3 ± 4.2	0.616	.425
9 mo	9.8 ± 4.0	7.3 ± 3.8	6.3 ± 2.9	0.833	.346
12 mo	9.1 ± 3.9	6.7 ± 2.8	5.4 ± 2.1	1.129	.217
18 mo	5.6 ± 1.9	4.3 ± 1.5	4.6 ± 1.4	0.371	.622
24 mo	4.4 ± 1.4	3.2 ± 1.3	2.9 ± 1.1	0.288	.732
**Presentation time of AKP (%**)					
3 mo	5 (45.45%)	2 (50%)	2 (40%)	0.656	.428
6 mo	2 (18.18%)	1 (25%)	1 (20%)	0.434	.472
9 mo	1 (9.09%)	0	1 (20%)	0.624	.421
12 mo	1 (9.09%)	1 (25%)	1 (20%)	0.583	.466
18 mo	1 (9.09%)	0	0	1.838	.279
24 mo	1 (9.09%)	0	0	1.271	.228

AKP = anterior knee pain.

### 3.4. Comparison of postoperative clinical outcomes

There were no significant difference in Oxford knee score, ROM, patellar score, KSS and ADL score among 3 groups. Compare with group T, the patient satisfaction score was significantly better (TPR: t = 2.651, *P* = .006; TPD: t = 2.178, *P* = .069) and VAS score was significantly lower (TPR: t = 2.636, *P* = .008; TPD: t = 1.979, *P* = .019) at 3 months postoperatively but not at the later follow-up time points in group TPR and TPD (Table 4).

**Table 4:** Comparison of postoperative clinical outcomes at different follow-up time among 3 groups.

Variables	Group T (n = 67)	Group TPR (n = 66)	Group TPD (n = 69)	F value	*P* value
**Oxford knee score**					
3 mo	47.9 ± 6.4	48.1 ± 6.2	48.2 ± 6.3	0.878	.324
6 mo	48.5 ± 6.1	48.7 ± 5.9	48.9 ± 5.7	0.467	.426
9 mo	48.9 ± 6.2	49.3 ± 6.0	49.5 ± 5.9	0.439	.518
12 mo	49.2 ± 6	49.6 ± 6.2	49.7 ± 6.3	0.372	.663
18 mo	49.6 ± 5.9	49.9 ± 6.2	49.8 ± 6.3	0.175	.821
24 mo	49.6 ± 6.2	49.9 ± 6.1	50.0 ± 6.1	0.208	.795
**ROM**					
3 mo	126.1 ± 11.3	126.9 ± 10.9	126.4 ± 11.3	0.483	.412
6 mo	127.5 ± 9.5	127.9 ± 9.3	127.8 ± 9.2	0.430	.538
9 mo	127.9 ± 9.3	128.2 ± 9.2	128.1 ± 9.3	0.398	.599
12 mo	128.8 ± 9.1	128.9 ± 9.3	128.8 ± 9.1	0.391	.622
18 mo	129.3 ± 8.9	129.3 ± 9.0	129.1 ± 8.9	0.380	.637
24 mo	129.5 ± 9.1	129.6 ± 9.2	129.8 ± 9.1	0.329	.728
**Patellar score**					
3 mo	25.3 ± 2.2	25.7 ± 2.6	25.6 ± 2.6	0.385	.632
6 mo	25.9 ± 2.4	26.1 ± 2.4	26.2 ± 2.3	0.403	.598
9 mo	25.9 ± 2.3	26.0 ± 2.3	26.3 ± 2.3	0.448	.479
12 mo	26.0 ± 2.2	26.2 ± 2.2	26.4 ± 2.1	0.424	.586
18 mo	26.1 ± 2.2	26.3 ± 2.2	26.4 ± 2.2	0.394	.620
24 mo	26.2 ± 2.2	26.3 ± 2.1	26.4 ± 2.1	0.365	.654
**KSS**					
3 mo	41.8 ± 7.8	42.9 ± 8.6	43.0 ± 8.8	0.801	.377
6 mo	42.5 ± 7.6	43.3 ± 7.6	43.2 ± 7.5	0.483	.419
9 mo	42.9 ± 7.5	43.7 ± 7.2	43.9 ± 6.9	0.454	.468
12 mo	43.3 ± 7.2	44.9 ± 7.1	44.8 ± 7.1	0.385	.634
18 mo	43.9 ± 7.0	45.4 ± 7.3	45.9 ± 6.8	0.353	.629
24 mo	44.8 ± 6.9	46.2 ± 7.4	46.4 ± 7.3	0.341	.698
**ADL score**					
3 mo	98.7 ± 2.3	98.9 ± 2.4	99.0 ± 2.5	0.802	.357
6 mo	98.9 ± 2.1	99.3 ± 1.8	99.2 ± 2.3	0.667	.396
9 mo	99.2 ± 2.0	99.5 ± 1.9	99.4 ± 2.1	0.455	.432
12 mo	99.4 ± 2.1	99.7 ± 1.8	99.7 ± 1.7	0.413	.589
18 mo	99.5 ± 1.7	99.8 ± 1.9	99.9 ± 1.9	0.440	.517
24 mo	99.5 ± 1.8	99.8 ± 1.9	99.9 ± 1.8	0.374	.658
**Satisfaction score**					
3 mo	11.1 ± 0.9	13.9 ± 0.8	13.8 ± 0.8	63.16	.038
6 mo	14.3 ± 0.5	14.6 ± 0.3	14.7 ± 0.2	0.461	.429
9 mo	14.4 ± 0.5	14.3 ± 0.5	14.8 ± 0.5	0.332	.724
12 mo	14.7 ± 0.3	14.8 ± 0.2	14.8 ± 0.3	0.202	.761
18 mo	14.8 ± 0.4	14.9 ± 0.3	14.9 ± 0.2	0.135	.846
24 mo	14.8 ± 0.3	14.9 ± 0.2	14.9 ± 0.2	0.113	.872
**VAS**					
3 mo	16.5 ± 2.3	11.8 ± 1.9	11.6 ± 1.8	113.41	.017
6 mo	6.9 ± 1.2	6.3 ± 1.3	6.1 ± 1.4	0.841	.337
9 mo	3.0 ± 0.9	2.9 ± 0.8	2.9 ± 0.8	0.636	.413
12 mo	1.5 ± 0.6	1.5 ± 0.5	1.4 ± 0.5	0.412	.588
18 mo	1 ± 0.3	0.9 ± 0.4	0.9 ± 0.3	0.195	.779
24 mo	0.8 ± 0.3	0.9 ± 0.4	1 ± 0.2	0.144	.818

ADL = activities of daily living, KSS = knee society score, ROM = range of motion, VAS = visual analog scale.

## 4. Discussion

More and more patients choose TKA to treat end-stage KOA, but with the increase of the number of operations, surgery related complications are also increasingly prominent, such as the occurrence of postoperative AKP, postoperative infection and prosthesis life, especially the residual postoperative knee pain, which puzzles surgeons and scholars, and also seriously affects the satisfaction of patients with surgery.^[13]^ In terms of incidence, postoperative AKP occurs between 2% and 25% of the time. Postoperative complications have also been the subject of many attempts by surgeons and scholars and they are still searching for effective ways to reduce AKP.^[14]^ Hence, it is of great clinical significance to explore the prevention and treatment strategy of AKP after TKA. In the present study, we found both PD and PR can effectively reduce the degree and incidence of AKP after TKA, and promote the recovery of knee function. Compared with PR, PD shows better effect.

TKA has been widely used in the treatment of KOA and rheumatoid arthritis, and a large number of patients receive this surgery every year. How to reduce the reoperation rate of replacement, reduce postoperative complications and avoid postoperative revision is an urgent problem to be solved at present. AKP is a common complication after TKA, which seriously affects the effect of surgical treatment and the postoperative quality of life of patients. Although the cause of postoperative AKP is not very clear at present, studies have confirmed that postoperative TKA may come from the following aspects: The high stress of patellofemoral joint leads to the increase of subchondral intraosseous pressure, and the abnormal patellar trajectory leads to the pathological changes of soft tissue around the patella, followed by patellar dislocation or inclination; The nerve of patella and its surrounding soft tissue is highly distributed, especially in the anterior knee soft tissue of degenerative cartilage, and the pain afferent fibers of substance P are widely distributed, which is easy to induce pain.^[15]^

Early knee arthroplasty does not include PR, because the activity of the prosthesis is anatomically mismatched with the autologous patella. Since 1955, the first PR prosthesis designed by mckeewer came out, people began to develop patellar prosthesis, and found that PR can reduce the incidence of postoperative patellofemoral joint pain, while reducing the incidence of reoperation, so PR was pursued by many mathematicians at that time.^[16]^ However, with the application of patellar prosthesis, the patella may be cut, which leads to related complications after PR, including patellar fracture, dislocation, septic osteonecrosis, prosthesis wear, loosening, knee extension device fracture and so on. Therefore, there are voices of opposition: without PR, good results will also be achieved and fewer complications will occur.

A large number of clinical practices have shown that, due to the changes in the existing mechanical properties and mucosa of the joint interface after TKA, the patella has lost its original motion performance.^[17]^ If the patella is not replaced, it may cause wear of the patella and femoral implants, leading to adverse consequences.^[17]^ Serious cartilage erosion is often found in the second operation. Those who do not support PR believe that the patella of the patient is more in line with physiology and anatomy, and PR also brings other complications. In addition, some patients with normal bone mass, no abnormal articular cartilage, good patellofemoral joint movement performance and no patellar disease can be cured without PR surgery.^[18]^ Combining the 2 points of view, those who support selective PR should consider various factors to selectively replace the patella. Although it has been controversial whether PR is necessary for the first TKA surgery, many studies have confirmed that PR can effectively reduce the incidence of anterior knee pain. In this study, we found that compared with conventional TKA, the incidence of AKP and the degree of AKP 3 months after PR in patients with VAS scores were significantly reduced, patient satisfaction was significantly improved, and there was no change in knee function after operation, which was consistent with the results of previous studies.

The purpose of PD is to block nerve innervations to the vastus medialis and the vastus lateralis in order to reduce AKP after TKA. Although the effect of this method to reducing forward anterior knee pain after TKA has been controversial, the mid-term effect is definitely.^[19]^ The innervation of the patella comes from the branches of the medial and lateral femoral muscles. The peripheral nerves can be damaged by electrocoagulation around the patella, thereby cutting off the transmission of pain. At the same time, it can heat and tighten the soft tissue during the burning process around the patella, which can effectively prevent the incarceration of soft tissue, so as to reduce pain and achieve the purpose of treatment. Professor Thiengwittayaporn carried out a 2-year follow-up study on 241 patients who under unilateral TKA. The results showed that AKP incidence and intensity were significantly lower, patient satisfaction scores were significantly better at 3 months after surgery in group PD. There were no significant differences between the 2 groups in Oxford knee score, ROM, Patellar score, KSS and ADL score.^[12]^ Two meta-analysis from Xie et al^[19]^ (including 374 articles and 898 patients with TKA) and Duan et al^[20]^ (including 37 studies and 1641 patients with AKP) showed that peripheral PD could effectively reduce the incidence of AKP and VAS score within 12 months after surgery. In this study, we found that compared with conventional TKA, the incidence of postoperative AKP, the degree of AKP and VAS score in patients receiving PD 3 months after TKA were significantly reduced, and the patient satisfaction was significantly improved, while the knee function remained unchanged after operation, which was consistent with the results of Thiengwittayaporn et al:

Based on the above advantages of PR and Pd on APK after TKA, we further compared PR and PD. The results showed that the intensity of AKP was significantly better at 3 months after TKA in group TPD, while there was no significant difference in other indicators. The reason may be that the aseptic loosening and subluxation of the inserted patellar prosthesis increase the degree of AKP.

It is undeniable that there are still some limitations in the present study. Firstly, the single center and prospective analysis design leads to some selection bias of the data; secondly, due to the long-time span of patients’ inclusion, surgical techniques were improved in the late stages, which may have an impact on data analysis.

In conclusion, our study indicated that PD and PR were significantly effective in relieving the incidence and intensity of AKP and improving patient satisfaction in TKA at 3 months after TKA. Additionally, PD is more effective on alleviating AKP than PR.

## Acknowledgments

The authors would like to thank all the members of department of orthopedics, Cangzhou Hospital of Integrated TCM-WM for their great help and support.

## Author contributions

Xiaohui Ji designed the study and drafted the initial manuscript; Xiaodan Huang and Yingying Zhang coordinated and supervised data collection. Ming Zhao and Yaming Liu collected and analyzed data. Yanxin Cheng reviewed and revised the manuscript.

**Conceptualization:** Xiaohui Ji.

**Data curation:** Xiaohui Ji, Yingying Zhang, Yaming Liu.

**Formal analysis:** Xiaodan Huang, Yingying Zhang, Yaming Liu.

**Funding acquisition:** Yanxin Cheng.

**Investigation:** Xiaodan Huang, Yanxin Cheng.

**Methodology:** Yanxin Cheng.

**Resources:** Ming Zhao.

**Software:** Ming Zhao.

**Supervision:** Ming Zhao.
